# “When the MRI Is Normal but the Bladder Isn’t: A Rare Case of Sacral Zoster–Associated Elsberg Syndrome”

**DOI:** 10.51894/001c.164582

**Published:** 2026-07-31

**Authors:** Austin Boots, Jash Mody, James Robinson, Myles Jerrett

**Affiliations:** 1 Henry Ford - Wyandotte

## 68

### Introduction

Herpes zoster reactivation involving the sacral dermatomes can rarely result in autonomic dysfunction known as Elsberg syndrome, a form of herpes virus–associated lumbosacral radiculitis. This condition is extremely uncommon, accounting for approximately 5–10% of acute lumbosacral radiculitis cases and less than 1% of herpes zoster complications. Because symptoms may mimic emergent spinal pathology such as cauda equina syndrome, prompt recognition is essential to avoid delayed treatment and potential long-term neurological dysfunction.

### Case Description

We present a case of a 62-year-old male with a history of chronic low back pain and type 2 diabetes mellitus who presented to the emergency department with pain in the gluteal folds and two days of progressive urinary retention and fecal incontinence. During the index visit, the patient was found to have a vesicular rash in the S2 dermatomal distribution. He reported multiple episodes of fecal incontinence without awareness and difficulty initiating urination and was found to have a post-void residual of 490 mL. Although he denied saddle anesthesia, recent spinal trauma, lower extremity weakness, or sensory deficits, there remained significant concern for cauda equina syndrome, and emergent MRI of the entire spine with and without contrast was obtained. Fortunately, imaging demonstrated no spinal cord compression, radiologic evidence of cauda equina syndrome, or cord signal abnormalities. Mild degenerative changes were noted and were considered likely incidental. In the setting of recent sacral herpes zoster and acute urinary retention with fecal incontinence, the presentation was most consistent with Elsberg syndrome. The patient was treated with acyclovir and high-dose methylprednisolone and admitted for neurological evaluation.

### Conclusions

This case highlights a rare cause of acute neurogenic bladder and bowel dysfunction and underscores the importance of recognizing sacral herpes zoster as a potential mimic of cauda equina syndrome when imaging fails to demonstrate compressive pathology.

